# Treating an intramuscular abscess following toothpick injury in a diabetic patient: A case report and literature review (CARE Complaint)

**DOI:** 10.1097/MD.0000000000018159

**Published:** 2019-11-27

**Authors:** Yu-Cheng Cheng, Po-Yu Liu, Sung-Yuan Hu

**Affiliations:** a Division of Endocrinology & Metabolism, Department of Internal Medicine, Taichung City, Taiwan; b Division of Infectious Disease, Department of Internal Medicine, Taichung Veterans General Hospital, Taichung City; c Department of Nursing, Shu-Zen Junior College of Medicine and Management, Kaohsiung City; d Rong Hsing Research Center for Translational Medicine, National Chung Hsing University; e Department of Emergency Medicine, Taichung Veterans General Hospital; f School of Medicine; g Institute of Medicine, Chung Shan Medical University; h Department of Nursing, College of Health, National Taichung University of Science and Technology; i Department of Nursing, Central Taichung University of Science and Technology, Taichung City, Taiwan.

**Keywords:** diabetes mellitus, intramuscular abscess, surgical debridement, toothpick injury

## Abstract

**Rationale:**

Toothpick puncture (TPP) is a penetrating injury that can result in bringing pathogens to the deep space. Such penetrating wounds are typically of pinpoint size with initial symptoms appearing subtle. Consequently, the injury itself is often neglected by patients, or is not detected during physical examinations by medical doctors. Reported complications from such injuries include osteomyelitis and septic arthritis, mostly due to delayed treatment.

**Patient concerns:**

A diabetic patient aged 83-year-old presented a 2-day history of skin redness, swelling, and tenderness over his forearm following a TPP a week earlier. Laboratory investigations showed leukocytosis with neutrophilic predominance and a high level of C-reactive protein. Before his operation, cultures of aspirated fluid from the injured site revealed the presence of *Streptococcus anginosus*, *Streptococci viridans*, *Prevotella intermedia*, and *Pavimonas (Peptostreptococcus) micra*.

**Diagnosis:**

Intramuscular abscess associated with toothpick injury.

**Interventions:**

Surgical irrigation with debridement and adjunctive antibiotics of ceftriaxone and clindamycin were given with a satisfactory response. Cultures of debrided tissue showed the presence of *P intermedia* and *P (Peptostreptococcus) micra*.

**Outcomes:**

A split-thickness skin graft was done. Patient was discharged on the 30th postoperative day.

**Lessons:**

Toothpick injury, initial symptoms of which are subtle, can in some cases, lead to serious complications especially when managements are delayed. In such situations (including the present case), surgical irrigation and debridement are administrated for the eradication of infections, removal of potentially retained toothpick, and tissue cultures analyzed. Adjunctive antibiotics is recommended to combat both the aerobic and anaerobic microorganisms of the gastrointestinal tract, skin surface, and oral cavity.

## 1 Introduction

A 1-year survey in the United States reported 8176 toothpick-related injuries (TRIs) in which 76% involved extremities and trunk.^[1]^ Toothpick punctures (TPPs) can lead to infections from polymicrobial oral flora, in mixed infections by both aerobic and anaerobic bacteria.^[2]^ TRIs are usually neglected by the affected subjects, because the wounds are seemingly small and often innocuous with subtle initial symptoms. Such injuries, especially with delayed management, can lead to serious complications. We reported here a case of a diabetic patient presented with a deep space infection (intramuscular abscess, IMA) after inflicting injury from a TPP. The patient recovered well after our treatments with surgical irrigation and debridement with adjunctive antibiotics, and finally with split-thickness skin grafts.

## 2 Case report

A male patient of 83-year-old had a type 2 diabetes mellitus (DM) with previous diagnosed (>20 years ago) complications of nephropathy and retinopathy, primary hypertension, and dyslipidemia. He came to our emergency department (ED) recently with a 2-day history of erythema, swelling, and tenderness felt over the left forearm. A week earlier, his left distal forearm was reportedly punctured by a used toothpick. At the ED, he complained of subjective fever and chills as well as severe forearm pain and swelling, which prompted his visit to our hospital.

Vital signs at presentation were the following: body temperature 38.0°C, blood pressure 144/74 mm Hg, pulse rate 77/minute, and respiratory rate 18/minute. Physical examination revealed a tender lesion over the left distal forearm with erythematous changes, swelling, and local heat. However, we found no signs of an open wound, bullae formation, or hemorrhagic changes. On palpitation, subcutaneous liquid accumulation was noted over the area of his left distal forearm. Aspirating the accumulated subcutaneous fluid revealed a straw-color liquid, a sample of which was sent for culture study. Empirical antibiotics with ampicillin/sulbactam were first administered intravenously. A complete blood count revealed 12,710 leukocytes/mm^3^ with neutrophilic predominance. The level of C-reactive protein was 23.38 mg/dL. Serum electrolytes were normal, but his blood level of glucose was 321 mg/dL and hemoglobin A1c 8.7 mmol/mol. Serum creatinine level was 3.49 mg/dL. Urinalysis results were normal. Culture of the aspirated fluid sample showed the presence of polymicrobial organisms, including *Streptococcus anginosus*, *Streptococci viridans*, *Prevotella intermedia*, and *Pavimonas (Peptostreptococcus) micra*.

**Figure 1:**
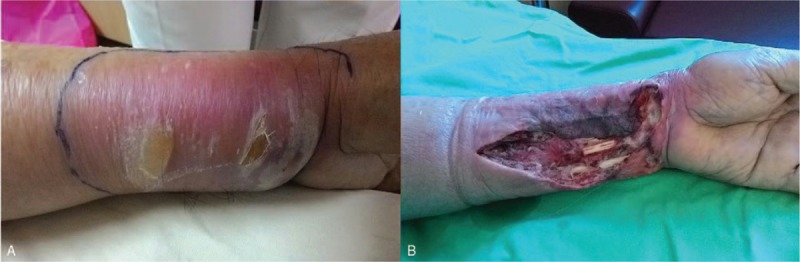
Preoperative photograph showing the area of swelling and erythema on the left forearm (A) of the patient after toothpick puncture injury a week earlier. Surgical debridement revealing intramuscular abscess and necrotic tissues (B).

On the 3rd day of admission, the erythematous changes progressed over his left forearm with increasing tenderness. Necrotizing fasciitis was highly suspected. We shifted the choice of antibiotics to ceftriaxone and clindamycin. Due to his condition of chronic kidney disease, computed tomographic scan with contrast media was not performed. He was taken to the operating room for surgical debridement. Wound cultures confirmed *Prevotella intermedia* and *P (Peptostreptococcus) micra*. IMA of the left distal forearm was diagnosed during the operation (Fig. 1). After operation, wound dressing with vacuum-assisted closure was given with antibiotics maintained throughout his hospital stay. The formation of granulated tissues over his wound bed was observed on postoperative day 21. In order to cover the affected area of his left forearm, we performed a split-thickness skin graft, which was harvested from his left anterior thigh. His condition subsequently improved, and the patient was finally discharged on postoperative day 30.

## 3 Discussion

Searching with the key word “toothpick” on PubMed, we found a total of 325 citations, within which 8 (1 case series, 7 case reports) are on soft tissue infections of the extremities following injuries from TPPs.^[2–9]^Table 1 shows the results of the 15 relevant cases. Of these cases, the commonest infection is deep space infection, which developed into conditions like soft tissue abscess (n = 6),^[2,4–6]^ pyogenic tenosynovitis (n = 5) (2 cases complicated with abscess formation),^[2]^ osteomyelitis (n = 3),^[7–9]^ and septic arthritis (complicated with abscess formation) (n = 1).^[2]^ Only 2 cases were diagnosed of superficial infection and cellulitis (n = 2).^[2,3]^

**Table 1:**
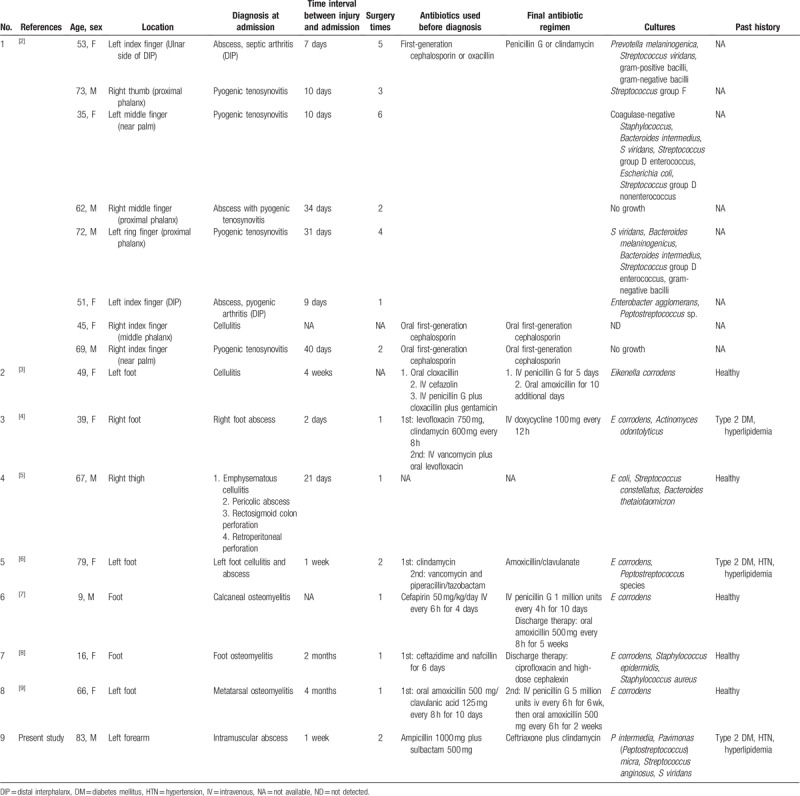
Literature reports of infection caused by toothpick penetration into limbs (7 case reports and 1 case series).

Generally speaking, TPP is a penetrating injury and may have the potential to bring pathogens to deep space (i.e., bone, tendon, and joint space). Injuries extended beyond the subcutaneous tissues are also of high risks of infection.^[10]^ In addition, the small penetrating wound from the toothpick is typically of pinpoint size and the initial symptoms are subtle, so the wound is often neglected by the affected subjects themselves, or undetected during physical examinations. The published cases often are characterized by complications including osteomyelitis and septic arthritis arising from delayed treatments. Intervals between injury and hospital admission lie between 7 and 40 days (average 17.6 days).^[2]^ Of the 15 cases shown in Table 1 (including a case series by Chang et al),^[2]^ the interval between injury and admission is between 2 days and 4 months, with a majority of them (n = 12) exceeding 1 week (Table 1).^[2–9]^

All 13 patients published with deep space infections caused by TPPs received surgical interventions.^[2,4–9]^ Surgical irrigation and debridement in conjunction with antibiotics are typically mandatory for deep space infections (e.g., abscess, necrotic soft tissue, tenosynovitis, septic arthritis, and osteomyelitis). Surgical debridement is generally warranted in the case of toothpick retention, because the retained foreign object of puncture is also a risk for infection, in line with reports on lacerating injuries.^[10]^ Surgical debridement allows the collection of tissue and fluid specimens for microbiological evaluation to identify the causative pathogens. As a general rule, since the penetrating wound from toothpick is minute, very limited amount of tissue can be collected for pathogen cultures. It is worth to point out that findings from superficial cultures of the wounds should be interpreted with caution, since cultures of superficial tissues do not always correlate in results with those of the deeper tissues.^[11–13]^ Pathogens are identified in tissue cultures collected during surgical irrigation and debridement in 12 of the 13 published cases on deep space infections.^[2,4–9]^ In conclusion, surgical intervention should be considered for infections caused by TPPs. As to the best time to apply the surgical intervention, studies published are too limited to provide a good recommendation.^[14]^

TPPs can be associated with infections by polymicrobial oral flora, with mixed aerobic and anaerobic infections reaching up to 71.4%.^[2]^ Of the 15 cases found in our literature search (including 8 cases by Chang et al,^[2]^ most of them (n = 8) have polymicrobial flora infections,^[2,4–6,8]^ and the anaerobic microorganisms, which are mostly oral flora, are identified in 11 cases.^[2–9]^*Eikenella corrodens* is the most common anaerobic microorganism (n = 6). *E corrodens* is a commensal of the human mouth and upper respiratory tract. With invasive infections of *E corrodens*, polymicrobial infections occur in 65% of the patients. Head and neck are the commonest infectious sites (56%) and nearly 2/3 of the patients (63%) have preexisting diseases, especially malignancy of head and neck.^[15]^ The disease condition also leads to infections in patients with insulin-dependent DM and intravenous drug users who might lick their needles (“needle-licker's osteomyelitis”).^[16]^ In some cultures (n = 6), anaerobic pathogens coexist with aerobic microorganisms such as *Escherichia coli*, *Streptococcus* group D, and *Enterobacter agglomerans* (which are common flora in gastrointestinal tract), *Streptococcus viridians* and *Streptococcus constellatus* (which are common oral flora), *Staphylococcus epidermidis* and *Staphylococcus aureus* (which are common flora on the skin surface).^[2,5,8]^

Generally speaking, toothpicks can have serious consequences similar to those following human bites. Infections of human bites are associated with alpha-hemolytic *Streptococci*, *S aureus*, *E corrodens*, *Haemophilus* species, and anaerobic bacteria (as in over half of the cases).^[17]^ Drugs, not able to suppress *E corrodens*, should be avoided. Such agents include the first-generation cephalosporins, macrolides, clindamycin, and aminoglycosides. Therefore, the treatment with amoxicillin/clavulanate, ampicillin/sulbactam, or ertapenem is recommended. If patients also have a history of hypersensitivity to β-lactams, the prescription of a fluoroquinolone (such as ciprofloxacin or levofloxacin) plus metronidazole, or moxifloxacin as a single agent is recommended.^[18]^ In patients with DM, the known mechanisms of defective immune defense include impaired tissue perfusion, chemotaxis, phagocytosis, and decreased bactericidal activity of neutrophils. Such weakness immunity results in sepsis and severe infections, like IMA.^[19,20]^ To treat infections caused by TRIs, coverage of both aerobic and anaerobic pathogens (including *E corrodens*) is recommended since we found in our patient, infectious pathogens similar to those found with human bites.

## 4 Conclusion

TRIs may have subtle initial symptoms which are easily neglected due to the seemingly small innocuous wound. Nonetheless it can lead to serious complications in delayed managements. Deep space infections should be considered. Therefore, surgical irrigation and debridement are the choice of treatment, in addition to collecting specimens for reliable cultures, and the removal of potentially retained toothpick. Adjunctive antibiotics should be considered to cover both aerobic and anaerobic microorganisms, including those floras at the gastrointestinal tract, skin surface, and oral cavity.

## Acknowledgments

In the clinical diagnosis and management of this patient, authors are grateful for the tremendous efforts of the emergency resuscitation team, the radiological technicians, and the help at the surgical intensive care unit.

## Author contributions

**Data curation:** Yu-Cheng Cheng, Po-Yu Liu, Sung-Yuan Hu.

**Investigation:** Yu-Cheng Cheng, Po-Yu Liu.

**Writing – original draft:** Yu-Cheng Cheng, Po-Yu Liu.

**Writing – review & editing:** Sung-Yuan Hu.
